# Endobronchial Hamartoma Subtotally Occluding the Right Main Bronchus and Mimicking Bronchial Carcinoid Tumor

**DOI:** 10.1097/MD.0000000000003369

**Published:** 2016-04-18

**Authors:** Filippo Lococo, Carla Galeone, Luciano Lasagni, Cristiano Carbonelli, Elena Tagliavini, Roberto Piro, Luigi Zucchi, Giorgio Sgarbi

**Affiliations:** From the Unit of Thoracic Surgery (FL, CG, GS); Pulmonology Unit (LL, CC, RP, LZ); and Unit of Pathology (ET), Arcispedale Santa Maria Nuova-IRCCS, Reggio Emilia, Italy.

## Abstract

Hamartomas are very rarely identified as an endobronchial lesion.

Herein, we describe a peculiar case of a 55-year-old woman with persistent cough and increasing dyspnea and radiological detection of a solid lesion subtotally occluding the main right bronchus. Despite the radiological and radiometabolic (18-fluoro-2-deoxy-d-glucose positron emission tomography/computer tomography scan) features were highly suspected for bronchial carcinoid, the definitive diagnosis after endoscopic removal was indicative of an endobronchial hamartoma.

When considering differential diagnosis of an endobronchial lesion, the physicians should take firmly in mind such rare entity and, accordingly, bronchoscopy and bronchoscopic biopsy should be done as first step in management of all cases presenting with endobronchial lesions.

## INTRODUCTION

Differential diagnosis of endobronchial tumors encompasses benign (e.g., hamartoma and lipoma) and malignant lesions (e.g., bronchogenic carcinoma, bronchial carcinoid (BC), metastasis, mucoepidermoid, or adenocystic carcinoma), generally considering first the bronchogenic carcinoma in the differential diagnosis of malignant endobronchial lesions.

Hamartomas are rarely identified as an endobronchial lesion; in the largest review series that has been published (215 patients), only 1.4% of hamartomas had an endobronchial location, and most of them were not associated with respiratory symptoms.^1^ Herein, we describe a peculiar case of an endobronchial hamartoma (EH) subtotally occluding the right main bronchus and mimicking BC tumor.

**FIGURE 1:**
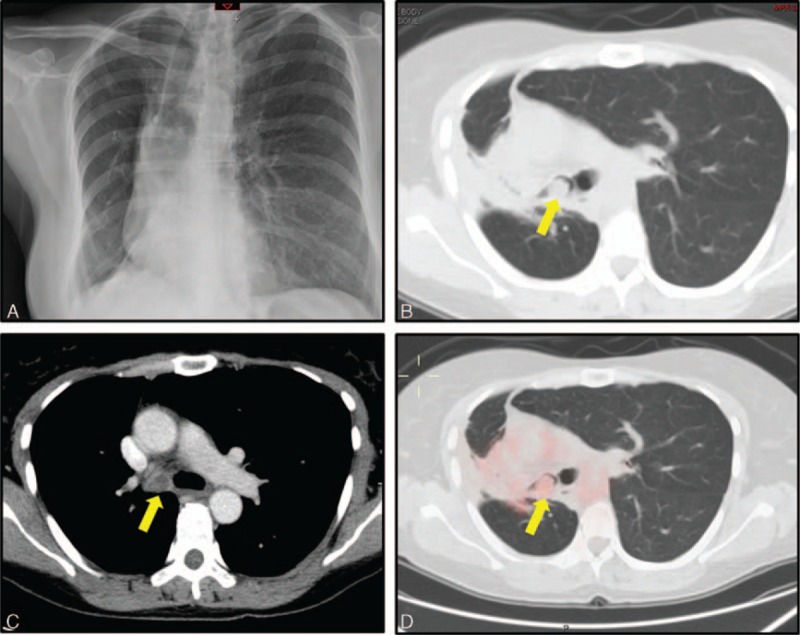
Radiological evaluation: chest x-ray (A) revealed a middle and lower pulmonary lobe collapse; contrast-enhanced CT-scan (B, C) showed the presence of a highly vascularized and round-shape solid lesion causing the subtotal obstruction of the main right bronchus; at ^18^F-FDG PET/CT scan (D) the lesion presented with a mild activity (SUVmax = 2.1), whereas no uptake was found in other areas. CT = computed tomography, ^18^F-FDG PET/CT = 18-fluoro-2-deoxy-d-glucose positron emission tomography/computed tomography.

A 55-year-old Caucasian woman with an unremarkable past medical history presented at Emergency Department for persistent cough and increasing dyspnea. Physical examination revealed decreased breathing sounds in the lower part of the right hemithorax. Chest x-ray (Figure 1A) revealed collapse of right middle and lower lobe of lung. A contrast-enhanced computed tomography (CT) scan revealed the presence of a solid endobronchial lesion subtotally occluding the main right bronchus (Figure 1B and C). Considering the radiological features highly indicative of BC (highly vascularized, round-shape well-defined lesion with slightly lobulated borders and punctate calcifications), an 18-fluoro-2-deoxy-d-glucose positron emission tomography/computed tomography (^18^F-FDG PET/CT) scan was planned for staging and diagnostic purposes. A mild uptake of the tracer was detected at the level of the endobronchial lesion (standard uptake value (SUVmax) = 2.1, Figure 1D), this confirming the initial hypothesis of endobronchial carcinoid tumor.

After multidisciplinary consultation, despite considering any risky procedure, an endoscopic removal was planned as first step approach with the double aim of solving the bronchial obstruction and obtaining a definitive pathological diagnosis. Despite we taken into account a 1,4,7,10-tetraazacyclododecane-N,N′,N″,N″’-tetraacetic acid (DOTA)-peptide PET scan evaluation for better characterizing the endobronchial lesion, we decided to perform directly the endoscopic removal of the lesion because the increasing dyspnea was becoming clinically significant (few violent episode of cough) and in consideration of the time required for scheduling such radiometabolic evaluation.

**FIGURE 2:**
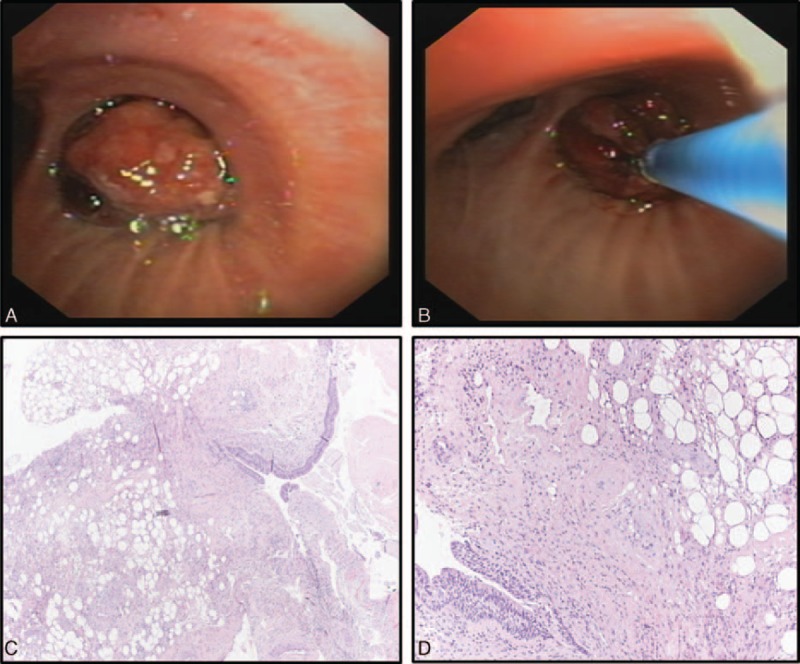
Pathological evaluation: the polypoid tumor was constituted by a mixture of fibrous connective tissue with jaline-cartilaginous, adipose, and fibrous components covered by bronchial respiratory epithelium (A, B).

Rigid bronchoscopy showed complete obstruction of the mid-portion of the right main bronchus caused by a polypoid yellowish lesion (Figure 2A); by using biopsy forceps and electrocautery snare, the lesion was accurately removed with no residual tissue (Figure 2B) and no significant bleeding occurred during the procedure.

Surprisingly, the definitive diagnosis was indicative of an EH (polypoid tumor containing mature cartilage with adipose and fibrous component covered by bronchial respiratory epithelium, Figure 2C and D). Considering the benign nature of the tumor, no further surgical procedures were performed.

A gradually and progressive improvement of the overall clinical conditions as well as of the radiological and endoscopic scenario was observed in the subsequent days.

After discharge, the patient underwent periodic endoscopic follow-up examinations and 12 months after operation, we observed no evidence of recurrence.

## COMMENT

Pure endobronchial neoplasm (defined as the tumor mainly involving the bronchial lumen) represents a rare entity with a histological diversity.^1^ Generally, malignant diseases are more common than benign ones with non-small-cell lung cancer remaining the most predominant malignant endobronchial histology in adults followed by BC tumor (more frequently reported in young people). Benign diseases are uncommon and, among these, EHs are a very entity with the largest series accounted for 43 EH cases.^2^ At careful revision of the pertinent literature, there aren’t reported specific radiological features of EH; generally, they appear as ovoid low-density endobronchial lesions,^3^ with a smooth edge, focal fat, or fat alternating with calcific foci (popcorn calcification).^4^ Such radiological findings appear substantially similar to those which may be found in BC tumors,^5^ this making the differential diagnosis based on radiological features only extremely challenging, as in the present case.

Treatment of the benign endobronchial tumor is almost debated; surgical approach could be considered only in very selected cases that are difficult to diagnose or remove by a bronchoscopic approach.^2^ On the contrary, some authors^6^ suggested the surgical approach as first treatment, considering that an endoscopic biopsy need to be weighed against bleeding risk especially when a BC is suspected and the lesion is located, as the present case, at the level of main bronchus.

Actually, in our opinion, the risk of bleeding in such cases could be remarkably minimized by just performing rigid bronchoscopy (instead of fiberoptic bronchoscopy). Moreover, as the present case clearly shows, a preliminary endoscopic removal may be extremely useful in all endobronchial lesions not only to solve the obstruction of the tracheobronchial tree but also to achieve a definitive diagnosis; this sometimes allows us to avoid unnecessary surgical resections for benign disease.^7^

In our case, the radiological features were indicative for a BC; the endobronchial lesion appeared as a round-shape lesion with slightly lobulated borders and presented punctate calcifications that may be seen in 39% of centrally located bronchial tumors.^5^

Moreover, the radiometabolic pattern (mild activity at 18-F FDG PET/CT scan) was also compatible with a well-differentiated BC tumor (so-called typical carcinoid), as reported in several studies.^8^ A DOTA-peptide PET scan could have been a very useful non-invasive diagnostic method. Indeed, considering the high accuracy in detecting BCs,^9^ a negative result at DOTA-peptide PET scan would be highly disproving a diagnosis of BC. Therefore, when feasible, such imaging tools should be considered in the diagnostic work-up examination of suspected BCs.

In conclusion, hamartoma may rarely occur as endobronchial tumor with a pattern of clinical symptoms and radiological/radiometabolic features that may mimic an endobronchial carcinoid tumor. Thus, a preliminary endoscopic approach may be extremely useful not only to solve the obstruction of the tracheobronchial tree but also to achieve a definitive diagnosis; this sometimes allows us to avoid unnecessary surgical resections.

## References

[1] Gjevre JA Myers JL Prakash UB Pulmonary hamartomas. *Mayo Clin Proc* 1996; 71:14–20. 8538225 10.4065/71.1.14

[2] Cosío BG Villena V Echave-Sustaeta J Endobronchial hamartoma. *Chest* 2002; 122:202–205. 12114359 10.1378/chest.122.1.202

[3] El-Kersh K Perez RL Gauhar U A 63-year-old man with a chronic cough and an endobronchial lesion. Diagnosis: endobronchial hamartoma. *Chest* 2014; 145:919–922.doi: 10.1378/chest.13-1965. 24687714 10.1378/chest.13-1965

[4] Suut S Al-Ani Z Allen C Pictorial essay of radiological features of benign intrathoracic masses. *Ann Thorac Med* 2015; 10:231–242.doi: 10.4103/1817-1737.160365. 26664560 10.4103/1817-1737.160365 PMC4652288

[5] Jeung MY1 Gasser B Gangi A Bronchial carcinoid tumors of the thorax: spectrum of radiologic findings. *Radiographics* 2002; 22:351–365. 11896225 10.1148/radiographics.22.2.g02mr01351

[6] Nussbaumer-Ochsner Y Rassouli F Uhlmann F Endobronchial lipoma mimicking bronchial carcinoid tumour. *Thorax* 2015; Mar 31. pii: thoraxjnl-2015-206923. doi: 10.1136/thoraxjnl-2015-206923, [Epub 2015 Mar 31]. 10.1136/thoraxjnl-2015-206923 25828429

[7] Zehani-Kassar A Ayadi-Kaddour A Marghli A Clinical characteristics of resected bronchial hamartoma. Study of seven cases. *Rev Mal Respir* 2011; 28:647–653.doi: 10.1016/j.rmr.2010.12.006. Epub 2011 Apr 16. 21645835 10.1016/j.rmr.2010.12.006

[8] Lococo F Cesario A Paci M PET/CT assessment of neuroendocrine tumors of the lung with special emphasis on bronchial carcinoids. *Tumour Biol* 2014; 35:8369–8377.doi: 10.1007/s13277-014-2102-y. Epub 2014 May 22. Review. 24850179 10.1007/s13277-014-2102-y

[9] Lococo F Perotti G Cardillo G Multicenter comparison of 18F-FDG and 68Ga-DOTA-peptide PET/CT for pulmonary carcinoid. *Clin Nucl Med* 2015; 40:e183–e189.doi: 10.1097/RLU.0000000000000641. 25608152 10.1097/RLU.0000000000000641

